# Pitfalls of Extensive Documentation in the Emergency Department

**DOI:** 10.31486/toj.19.0108

**Published:** 2020

**Authors:** Andrea Blome, Daohai Yu, Xiaoning Lu, Kraftin E. Schreyer

**Affiliations:** ^1^ Department of Emergency Medicine, Temple University Hospital, Philadelphia, PA; ^2^ Department of Clinical Sciences, Lewis Katz School of Medicine at Temple University, Philadelphia, PA

**Keywords:** *Clinical coding*, *documentation*, *topography–medical*

## Abstract

**Background:** The law mandates careful record-keeping in the emergency department, and clinical imperatives also support the value of complete and legible reports. A common assumption is that extensive documentation increases the yield of relative value units (RVUs) and higher levels of care, thereby maximizing reimbursement. However, overdocumentation presents certain risks, possibly impacts physician efficiency, and does not ensure that records are more readable and clinically useful. We examined the effect of increased documentation on actual reimbursement.

**Methods:** We conducted a 12-month productivity analysis of patients per hour (pt/h), RVUs per hour (RVU/h), amounts of monies billed, and amounts of monies collected for all full-time supervising physicians in a university emergency medicine training program.

**Results:** RVU/h vs pt/h yielded a positive linear relationship (R^2^=0.7571) and a strong correlation coefficient of 0.87. RVU/h vs revenue collection (amount actually paid) yielded a moderately positive linear relationship (R^2^=0.1752), with a correlation coefficient of 0.42. The relationship between pt/h and collections was weak (R^2^=0.0815), with a correlation coefficient of 0.29. A quartile comparison showed an inflection point, suggesting that after the third quartile, RVU/h did not appear to help generate significantly higher collections.

**Conclusion:** The data, while not definitive, suggest that overly extensive documentation may increase RVU totals but, after a point, does not reliably increase revenue generation.

## INTRODUCTION

Every emergency patient encounter requires documentation, and charting is expected to be thorough and accurate. Aside from being a legal document, the emergency department (ED) medical record has clinical and financial functions. Clinically, the medical record communicates vital information to providers—whether the inpatient team, an office-based physician, or the emergency medicine physician at successive visits. Medical records also form the basis of billing. Compensation is based on the work performed as determined by coding. Generally done by a third party, coding is the process of deconstructing the ED visit into a level of service and the procedures performed.

Relative value units (RVUs) represent an arbitrary measurement of physician work; they are measures of value based on the Medicare reimbursement formulae for physician services.^1^ RVUs extracted from medical records factor into the compensation that the physician contributes to the department. In the unique clinical setting of the ED, in which patients present unpredictably with varying degrees of acuity and complexity, RVUs track a confusing mix of cognitive and procedural efforts. Unlike their colleagues in the surgical and medical departments, ED physicians generally have no knowledge of how many RVUs will be generated for work with a particular patient. Not surprisingly, billing efforts on behalf of emergency physicians require trained billing specialists, either outsourced or in-house.

Different evaluation and management codes apply to the context of a patient encounter, such as in-hospital or outpatient visits, and factor into the level of service. The level of service ranges from 1 to 5, with an additional designation for critical care, and is determined by how much clinical information is documented in the history, review of systems, physical examination, and medical decision-making. In general, the more complex the visit, the higher the level of service.^2^ Whether the physician is a direct employee, a contractor, or a member of an independent group, documenting and billing for all work legitimately performed is imperative.^3^ Trainees are generally directed to document extensively.^4^ Supervising physicians may also be encouraged by administrators or billing services to provide appropriate documentation to qualify for the highest appropriate level of service.

The widespread use of electronic medical records allows for fast and easy documentation with prepopulated templates and phrases. Templated medical records are designed to efficiently capture required histories (present illness, past illnesses, social and family history, review of systems), objective findings, and data review to justify coding levels. Emergency medicine practice entails multitasking of clinical problems involving varying degrees of acuity and complexity. The relative ease of computerized charting can allow for careful review of systems pertinent to the chief complaint, and an unsuspected issue may shed light on the presenting complaint. For instance, a patient presenting with leg edema who is found to have pruritis on review of systems would suggest a liver pathology as the cause. Further, an incidental finding could lead to the discovery of an unrelated but significant pathology. For example, a patient presenting with ankle pain who is found to have unintentional weight loss on review of systems may suggest an undiagnosed malignancy. Thorough use of a review of systems may provide the physician with diagnostic acumen; however, an extensive review of systems may also lead to notation of system complaints that are not thoroughly investigated. Casual reference to incidental findings can be medicolegally problematic if the findings are not fully investigated or followed up.

What might be considered overdocumentation can also result in overbilling. Third-party payers—such as government agencies, insurance companies, health maintenance organizations, and employers—review emergency medicine group practices. A group with high levels of complex care billings could invite audits, resulting in paybacks and penalties. In rare cases, charges of fraud have been leveled. For instance, Prime Healthcare Services, Inc. agreed to pay $1.25 million to settle upcoding allegations regarding falsified information about patient comorbidities and complications.^5^ As medical billing companies process healthcare encounters into claims for insurance companies and patients, they can provide protection against potential penalties. To protect physicians and themselves, billing specialists may intentionally bill a medical record documented at a level 5 to a lower level. In other words, careful charting by a physician to justify a high level of service may be reimbursed at a lower level.^3^

Another consideration related to overdocumentation is that time spent charting may be at the expense of patient care. An observational study of office-based physicians showed that physicians spent 2 hours on the computer for every hour of patient time.^6^ Although some charting is often done after the scheduled end of shift, this study demonstrated that charting takes more clinical time than direct patient care.

Conceptually, given a cohort of ED patient encounters, opportunities for reimbursement may be missed when charting is done in the context of a busy ED. Again, conceptually, given the same cohort of emergency patients, there must be a level at which all possible billable issues have been captured, and further documentation, from the point of view of billing, would be overdocumentation.

While it is arguable to what degree extensively detailed charting may be clinically valuable, a common assumption is that adding more information to documentation will improve reimbursement. To what degree this assumption is accurate is the focus of this study.

## METHODS

We conducted a 1-year productivity analysis of all supervising physicians (n=39) across 3 hospitals in an urban, university emergency medicine training program located in Philadelphia, PA. The health system serves an area of the state in which 30% of the population lives below the federal poverty level, and the majority of the payers are government-funded programs such as Medicare and Medicaid.^7^

All supervising physicians work 8-hour shifts, with a range of 8 to 18 shifts per month. Physicians in training work during most shifts at all 3 sites. The department at the main university hospital is divided into 3 zones according to acuity. The other hospitals in the system each have 1 designated main ED and 1 minor care area. Minor care areas at all 3 sites are staffed by advanced practice providers. No advanced practice provider medical records were included in this analysis.

At the study institution, a third-party company that contracts with the health system handles billing. The electronic medical record generates coding levels that the hospital's coding department confirms. The medical records are then sent to the billing company for final review.

Data for each supervising physician were assessed for patients per hour (pt/h), RVUs per hour (RVU/h), amounts of monies billed, and actual reimbursement from billings. Ranges were averaged first by physician. Because physicians worked varying numbers of hours, we calculated percentages of reimbursement to billings to standardize the evaluation. The relationships between RVU/h vs pt/h, RVU/h vs revenue collected, and pt/h vs revenue collected were determined. The R^2^ value for each relationship was used to determine variance, and a correlation coefficient was also calculated. For trending purposes, physicians were ranked and subsequently divided into RVU quartiles based on billings and revenue collection percentages.

## RESULTS

**Figure 1.**
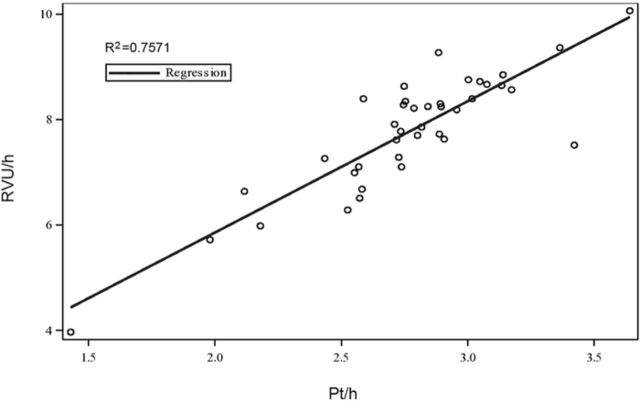
**Scatter plot demonstrating a strong linear relationship between patients seen per hour (pt/h) vs relative value units per hour (RVU/h) for each supervising physician. A trend line is included, with an R^2^ of 0.7571.**

Among the 39 physicians, pt/h ranged from a low of 1.3 to a high of 3.97. RVU/h ranged from a low of 3.97 to a high of 10.07. A scatter plot of pt/h vs RVU/h showed a strong, positive liner relationship (R^2^=0.7571) and a correlation coefficient of 0.87 (Figure 1).

**Figure 2.**
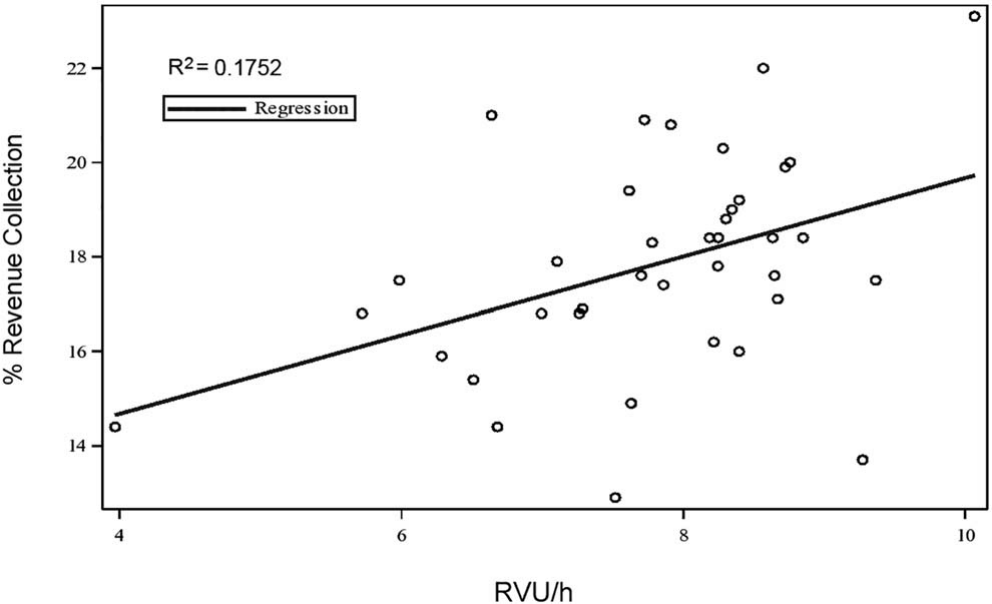
**Scatter plot of relative value units per hour (RVU/h) plotted against percent revenue collection demonstrating a moderate positive linear relationship, with an R^2^ of 0.1752.**

Percentages of actual revenue collected vs billings ranged from a low of 12.9% to a high of 23.1%. RVU/h vs revenue collection yielded a moderately positive linear relationship (R^2^=0.1752) with a correlation coefficient of 0.42, suggesting that adding more detail to documentation to produce higher RVUs may not translate to increased revenue (Figure 2).

**Figure 3.**
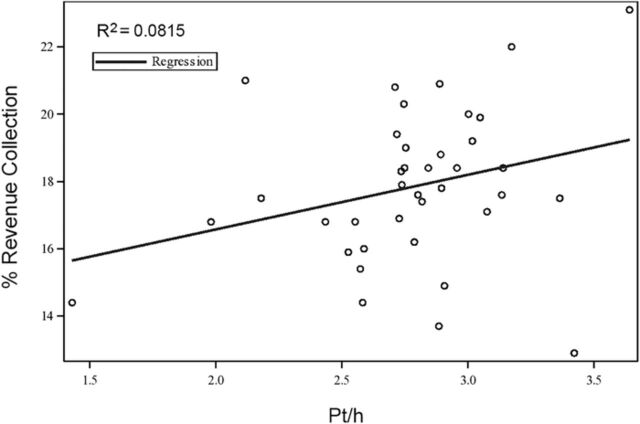
**Scatter plot of patients seen per hour (pt/h) vs percent revenue collection demonstrating a weak linear relationship, with an R^2^ of 0.0815, implying a very limited predictability of pt/hr for the percentages of actual revenue collection.**

The analysis of pt/h vs revenue collection yielded a weak relationship (R^2^=0.0815), with a correlation coefficient of 0.29 (Figure 3).

**Figure 4.**
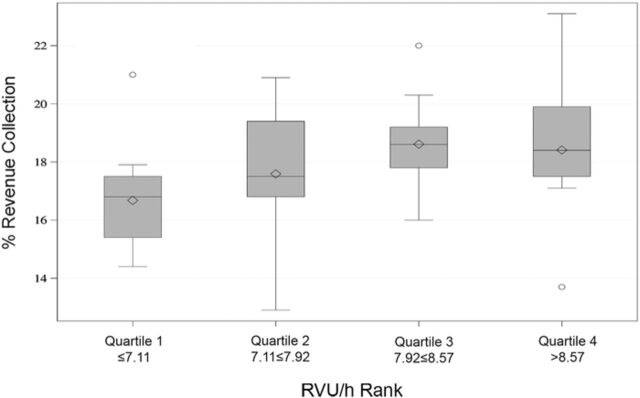
**Quartile graph of relative value units per hour (RVU/h) rank vs percent revenue collection demonstrating an increase in revenue collection until the third quartile.**

**Figure 5.**
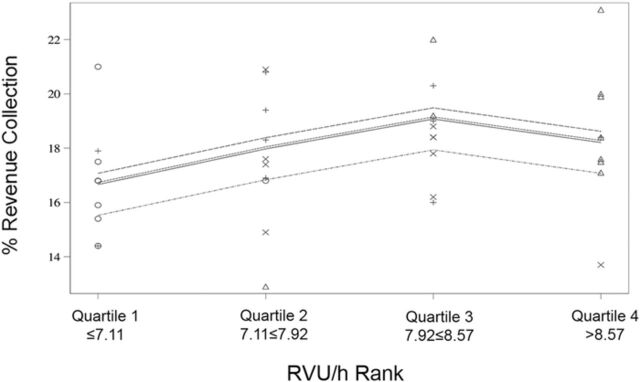
**Interaction plot of relative value units per hour (RVU/h) rank vs percent revenue collection demonstrating an inflection point after which percent revenue collection no longer increases.**

A quartile analysis and interaction plot comparing RVU/h to percent revenue collections showed an increase in RVU/h until the third quartile (Figures 4 and 5).

## DISCUSSION

This study suggests an inflection point at which documentation for more RVUs did not appear to generate significantly higher collections. Beyond this point, the ED physician may be documenting too much. While complex patients may need extensive documentation, for a range of patients, overdocumentation is not without hazard. Medicolegally, documentation of less clinically pertinent issues that are not fully investigated may be hard to defend, as can documentation of negative system reviews that are not matched by in-depth history taking. Clinically, more time spent documenting indicates less time spent with patients.^8^ Documentation of history-taken positives not apparently relevant to the immediate clinical problem could mandate special studies and procedures that could extend length of stay and impair patient flow.

Electronic medical records have made charting easier and quicker to accomplish, as well as provided ready access to complete and legible records. As with all technologies, computerized charting is not without its downsides and may sometimes lead to its own form of overuse.

One limitation of this retrospective observational study is that metrics for RVU/h, pt/h, billings, and revenue collections are precise but only for an institution with a poor payer mix. A similar study in a more affluent demographic might show greater variability in reimbursement yields. In addition, this study was conducted at a training university health system with training physicians rotating in all 3 hospitals in the system. The training physicians chart, but the supervising physician is responsible for the final documentation and can edit a trainee's charting. Time devoted to training physicians likely impacted throughput and documentation. Further, similar to the training physicians, the supervising physicians rotate among the 3 hospital sites. Because of the way treatment zones are apportioned, physicians have little opportunity to preferentially choose lower acuity patients who would require less treatment time. Advanced practice providers see the patients requiring minor treatment during the day and evening shifts but not overnight. The 5 nocturnists, therefore, see more lower acuity volume than the physicians working day and evening shifts. Seeing lower acuity patients on the overnight shift could allow physicians to treat more patients per hour which would impact the results of the analysis. For the remaining physicians, discrepancies in acuity should equalize over the course of a year. Scribes, who were employed in lower acuity zones and occasionally in minor care areas, may have impacted efficiency, but that was not analyzed in this study. Finally, the data were compiled for each physician for a 1-year time period, and more granular data, such as by shift, were not obtained.

## CONCLUSION

The importance of accurate and detailed medical documentation is inarguable. However, our data indicate an inflection point beyond which documentation did not appear to generate significantly higher collections. Documentation should, therefore, focus on important clinical information rather than on billing outcomes.

## Notes

This article meets the Accreditation Council for Graduate Medical Education and the American Board of Medical Specialties Maintenance of Certification competencies for Medical Knowledge, Systems-Based Practice, and Practice-Based Learning and Improvement.
